# Penile Embryology and Anatomy

**DOI:** 10.1100/tsw.2010.112

**Published:** 2010-06-29

**Authors:** Jenny H. Yiee, Laurence S. Baskin

**Affiliations:** Department of Urology, University of California, San Francisco, CA, USA

**Keywords:** penis, urethra, anatomy, embryology

## Abstract

Knowledge of penile embryology and anatomy is essential to any pediatric urologist in order to fully understand and treat congenital anomalies. Sex differentiation of the external genitalia occurs between the 7and 17 weeks of gestation. The Y chromosome initiates male differentiation through the SRY gene, which triggers testicular development. Under the influence of androgens produced by the testes, external genitalia then develop into the penis and scrotum. Dorsal nerves supply penile skin sensation and lie within Buck's fascia. These nerves are notably absent at the 12 o'clock position. Perineal nerves supply skin sensation to the ventral shaft skin and frenulum. Cavernosal nerves lie within the corpora cavernosa and are responsible for sexual function. Paired cavernosal, dorsal, and bulbourethral arteries have extensive anastomotic connections. During erection, the cavernosal artery causes engorgement of the cavernosa, while the deep dorsal artery leads to glans enlargement. The majority of venous drainage occurs through a single, deep dorsal vein into which multiple emissary veins from the corpora and circumflex veins from the spongiosum drain. The corpora cavernosa and spongiosum are all made of spongy erectile tissue. Buck's fascia circumferentially envelops all three structures, splitting into two leaves ventrally at the spongiosum. The male urethra is composed of six parts: bladder neck, prostatic, membranous, bulbous, penile, and fossa navicularis. The urethra receives its blood supply from both proximal and distal directions.

## PENILE EMBRYOLOGY

External genital differentiation into the male sex begins in the 7th week of gestation and is completed by 16–17 weeks[1,2,3]. Prior to this, between the 4th and 7th weeks, the mesodermal mesenchyme migrates to the cranial aspect of the cloacal membrane to form the genital tubercle. The cloacal membrane itself is composed of two layers: endoderm and ectoderm. The caudal portion of the cloacal membrane develops into urogenital folds. These structures are the precursors for external genitalia in both males and females[4], Fig. 1.

In those with a Y chromosome, the SRY gene signals the differentiation of primitive sex cords into testes by first signaling the development of Sertoli cells[5]. Sertoli cells then aid in the development of germ cells and Leydig cells[6] within the testes. Leydig cells produce testosterone, which is converted to dihydrotestosterone to induce external genitalia development.

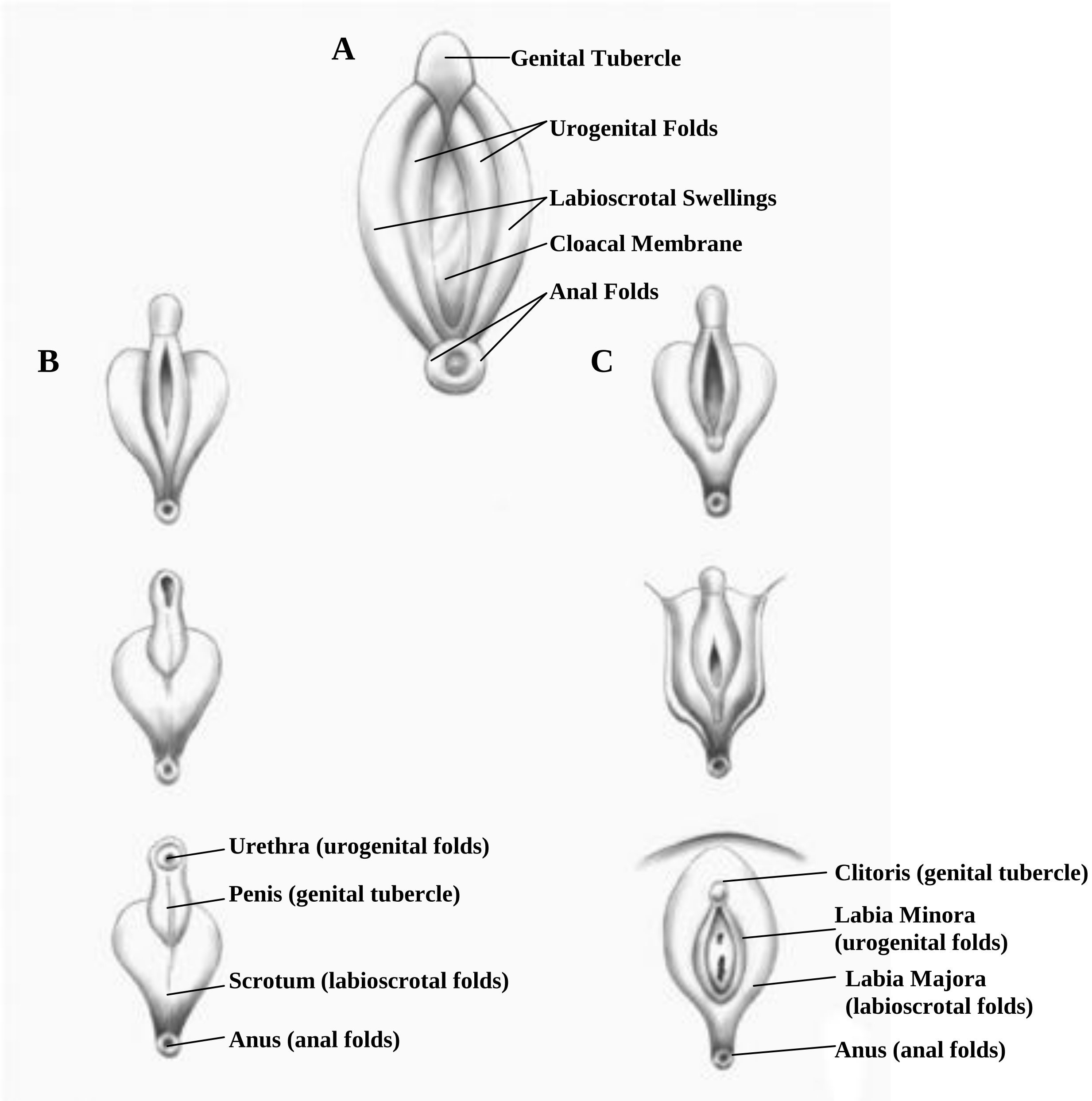

**FIGURE 1**. Human urogenital embryology. (A) Sex undifferentiated precursors prior to 7 weeks gestational age. (B) Male development in gestational weeks 7–17 from precursors shown in (A). (C) Female development in weeks 7–17 from precursors shown in (A).

Under the influence of androgens, in males the mesoderm of the genital tubercle enlarges to become the corpora cavernosa and glans penis. The endoderm tubularizes in a proximal to distal direction to form the penile urethra. The ectoderm develops into the penile skin and prepuce, which begins formation at 8 weeks. There is some debate as to whether the distal urethra is formed from tubularization of the endoderm, as with the proximal urethra, or from canalization of the ectoderm distally[2,7].

## PENILE ANATOMY

### Nerves

Penile innervation consists of the dorsal, cavernosal, and perineal nerves. Dorsal nerves arising from the pudendal nerves travel within Buck’s fascia, together with the dorsal arteries and veins, to supply sensation to penile skin[8], Fig. 2. Despite its nomenclature, it is important to note that the nerves do not lie directly in the dorsal midline, but rather extend from the 11 and 1 o’clock positions laterally to the junction of the cavernosa and spongiosum[9,10], Fig. 2. These nerves do not send perforators deep through the tunica albuginea to the corpora cavernosa[9]. There is a paucity of nerves at the 12 o’clock shaft position. Therefore, in correction of penile curvature, plication at the 12 o’clock position is the area least likely to result in nerve damage.

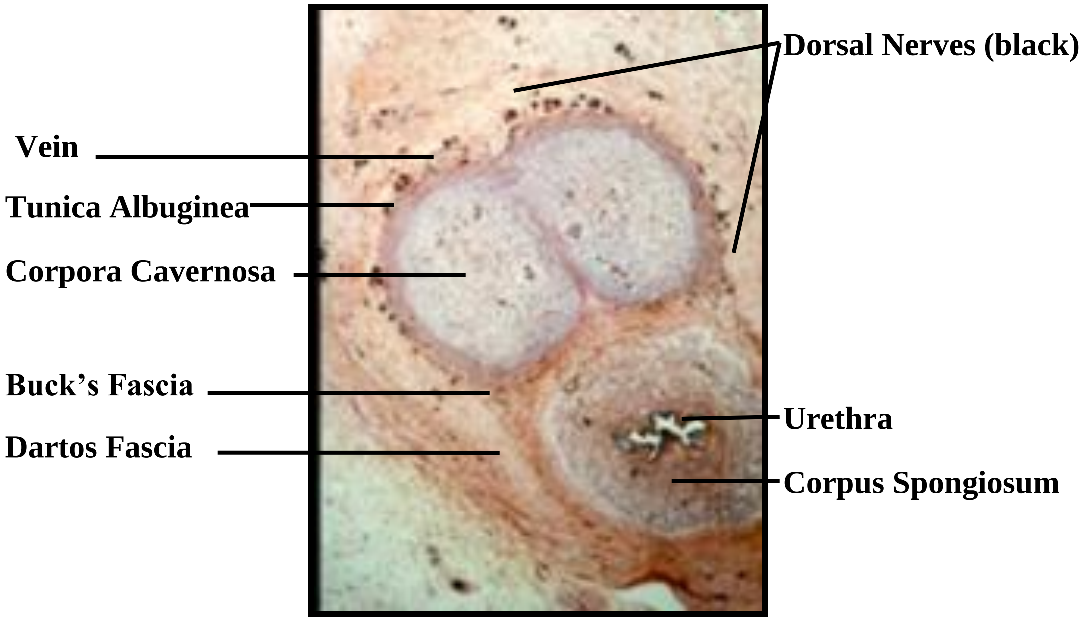

**FIGURE 2.** Histology of penile anatomy in cross-section. Note that the dorsal nerves start at the 11 and 1 o’clock positions of the corpora cavernosa and extend around laterally to the cavernosal-spongiosal junction. There is a notable paucity of nerves at the 12 o’clock position.

Like the dorsal nerves, the perineal nerves also arise from the pudendal nerve to supply the ventral shaft skin, the frenulum, and the bulbospongiosus muscle[11], Fig. 3. The cavernosal nerves arise from the autonomic pelvic plexus and travel along the periprostatic neurovascular bundle, well known to urologists performing radical retropubic prostatectomies. Underneath the pubic arch, the cavernosal nerves pierce through the corpora cavernosa[12], Fig. 4. Proximal to this point, the cavernosal and dorsal nerves lie within close proximity at the penile hilum and are thought to exchange signal communication, which may have implications on erectile function. As well, there are interactions between perineal and dorsal nerves laterally at the junction of the cavernosa and spongiosum along the penis, which may also have implications on erection and ejaculation[13].

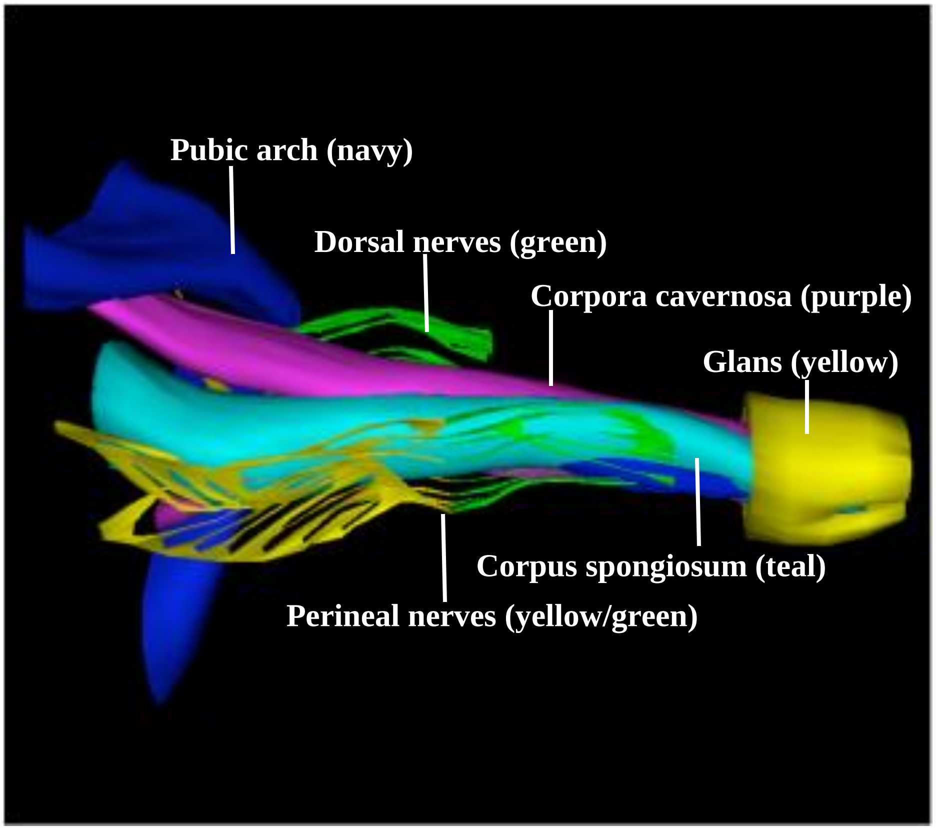

**FIGURE 3.** Ventral-lateral view: 3-dimensional reconstruction of penile anatomy.

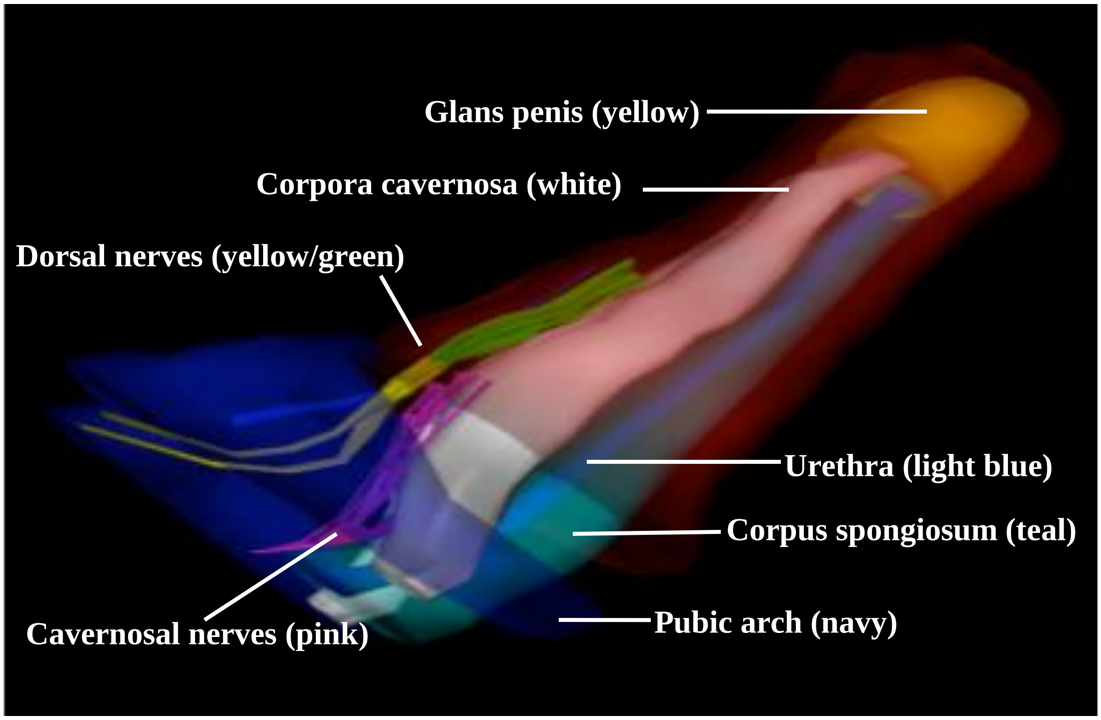

**FIGURE 4.** Dorsal-lateral view: 3-dimensional reconstruction of penile anatomy.

## Vasculature

### *Arteries*

There are three paired main arteries in the penis: cavernosal, dorsal, and bulbourethral. All three arise from a shared branch of the internal pudendal artery, which itself arises from the internal iliac artery. On each side, the first branching occurs at the bulb of the spongiosum external to the urogenital diaphragm forming the bulbourethral artery, which then lies at the 9 and 3 o'clock positions of the corpus spongiosum. Then the cavernous artery branches to penetrate the corpora cavernosa and the remainder of the artery continues as the deep dorsal artery. The deep dorsal artery causes glans enlargement during erection, whereas the cavernosal arteries cause corporal enlargement. All three arteries communicate distally near the glans to provide an extensive anastomotic network[8,14].

Penile skin derives its supply from a separate origin. Branches of the external pudendal artery supply the dorsal and lateral aspects of the penis, and branches of the internal pudendal artery supply the ventral penis and scrotum via the posterior scrotal artery[15]. These branches course in the Dartos fascia and enable pedicled skin flaps to be used in urethral reconstruction.

### *Veins*

Venous drainage is not analogous to arterial supply, unlike many other body systems. In contrast to the paired dorsal arterial system, there exists only one deep dorsal vein that runs alongside the dorsal arteries and nerves in Buck's fascia above the tunica albuginea. The deep dorsal vein receives drainage from the distal two-thirds of the corpora cavernosa via emissary veins and the corpus spongiosum via circumflex veins. Emissary veins are the veins that traverse obliquely through the tunica albuginea, allowing them to be compressed during erections for penile tumescence. The deep dorsal vein then drains to the periprostatic plexus[16].

Recently, a small pair of dorsal veins have been found that lie just deep to the deep dorsal vein, but above the tunica albuginea, which independently receive emissary vein drainage. These veins have been termed cavernosal veins, but do not lie within the corpora cavernosa[17]. Older literature refers to the cavernosal veins as short veins located in the triangle between the proximal crus that drain the proximal one-third of the corpora cavernosa. These veins join with the bulbourethral veins (which drain the proximal spongiosum) to lead into the internal pudendal vein. The penile skin drains via the superficial dorsal vein, which drains into the saphenous vein.

## Corpora Cavernosa and Glans

The proximal crus of the corpora cavernosa begin as separate structures spread apart under the ischium bilaterally and move medially under the pubis until they lie adjacent to each other. They are each enveloped in a fibrous tunica albuginea, which share a septum medially when the crus are adjacent. This septum is perforated, allowing for communication of the cavernosal sinuses in the penis. The distal tips of the corpora cavernosa are capped by the glans penis, which is itself a continuation of the corpus spongiosum that surrounds the urethra. Buck's fascia surrounds the paired corpora cavernosa and corpora spongiosum circumferentially. It splits into two leaves to surround the corpus spongiosum completely on the ventral aspect of the penis, see Fig. 2. An extension of Buck's fascia forms the suspensory ligament, which attaches the penis to the underside of the pubis. Buck's fascia is continuous with deep pelvic muscle fascia. Superficial to Buck's fascia is Dartos fascia, which is continuous with Scarpa's fascia of the abdomen and Dartos fascia of the scrotum[8,14].

## Urethra

The male urethra is divided into six parts: bladder neck, prostatic urethra, membranous urethra surrounded by external sphincter, bulbous urethra proximal to the ischiocavernosus muscle, penile/pendulous urethra distal to the ischiocavernosus muscle, and the fossa navicularis within the distal glans. The corpus spongiosum is erectile tissue akin to corpora cavernosa, but with a thinner tunica albuginea. The penile and bulbar urethra lie within the spongiosum. The penile urethra lies in a central location within the spongiosum, whereas the bulbar urethra lies eccentrically closer to the dorsal spongiosum prior to exiting dorsally to become the membranous urethra to join the prostate[15]. Whereas the condition known as “chordee” or penile curvature was once believed to result from fibrous bands near the urethra, no such fibrous tissue has been found in the penile urethra, even in severe cases of hypospadias[18]. Because of anastomotic communications between the dorsal arteries and the bulbourethral arteries, the urethra receives arterial supply from both distal and proximal directions. This enables complete transaction of the urethra without necrosis of the distal segment.
